# Long-Term Caregiving Impact and Self-Care Strategies in Family Caregivers of People with Neuropsychiatric Disorders: A Mixed-Method Study

**DOI:** 10.3390/diseases12110292

**Published:** 2024-11-14

**Authors:** Vanessa Sánchez-Martínez, Omar Cauli, Silvia Corchón

**Affiliations:** 1 Nursing Department, University of Valencia, 46010 Valencia, Spain; vanessa.sanchez@uv.es (V.S.-M.); silvia.corchon@uv.es (S.C.); 2 Frailty Research Organized Group (FROG), University of Valencia, 46010 Valencia, Spain; 3 Chair of Healthy, Active and Participative Aging, University of Valencia, 46010 Valencia, Spain

**Keywords:** long-term conditions, family caregiver, needs assessment, self-care strategies, neuropsychiatric disorders

## Abstract

Family caregivers of people with neuropsychiatric conditions are at risk of caregiver burden and declining health. The aims of this study were to identify the impact of caring on long-term family caregivers and their unmet needs and to explore their self-care strategies for achieving a successful caregiving experience. A mixed-method study was conducted using semi-structured interviews and a questionnaire in which standardized, self-reported measures of burden, health behaviors, sleep, and mental well-being were administered. Participants were family caregivers of people with neuropsychiatric disorders. Convenience sampling of 28 caregivers: 13 of people with mental health disorders (schizophrenia and bipolar disorder) and 15 with Alzheimer’s disease. Based on the analysis of the semi-structured interviews, data saturation was reached. Analysis of self-reported measures indicated that 32.1% of long-term caregivers had high caregiver burden, 64.3% had reduced quality of life, 39.3% had low sleep quality, 21.4% had low adherence to the Mediterranean diet, 50.0% had a physical activity below the recommendation, 42.9% had high anxiety symptoms, 35.7% had high depressive symptoms, and 71.4% had reduced self-care agency. Content analysis and statistical analysis were conducted. Two themes were identified: (1) the impact of long-term caregiving and unmet needs and (2) successful self-care strategies. Caregivers of people with Alzheimer’s disease spent less time doing physical activity, had higher caregiver burden, and poorer health-related quality of life. The negative impact of caregiving could be prevented/managed by assessing the individual’s circumstances for the development of cross-sectional self-care strategies involving physical, emotional, and social spheres.

## 1. Introduction

Long-term or chronic conditions (LTCs) are processes with a long duration and slow progression requiring continuous care and treatment [1], causing the most disabilities and deaths and extensive resource consumption for healthcare systems. Projections for the coming years suggest that this situation will become progressively more acute, creating a growing demand [2]. Living with an LTC is not an individual issue, but also a family affair [1], as in most cases the caring and living experience takes place within the family. Family caregivers are “non-professional persons who provide primary assistance with activities of daily living towards a dependent person in their immediate circle” [3]. Since up to 80% of all long-term care in Europe is provided by informal caregivers, family caregivers’ health, well-being, and quality of life are essential [3,4]. Maintaining quality of life and self-care among caregivers has repercussions for the caregivers themselves and impacts those receiving care and the entire healthcare system [5]. Family caregivers of people with CCs may present complex needs due to the impact of caring on their health and well-being. In a recent study, the unmet needs of 457 family caregivers living in English-speaking countries were analyzed through an online survey, and the authors concluded that over 70% of the participants needed (1) to reduce stress in their own lives, (2) to balance the needs of the person they cared for with their own, and (3) to look after their health, including eating and sleeping properly [6].

Among the CCs, neuropsychiatric disorders such as Alzheimer’s disease and severe mental disorders stand out not only because of their prevalence but also due to their potential impact on both the person affected and their family [4]. This impact is also expected to be more significant when the person presents behavioral symptoms. Alzheimer’s disease (AD) is the most common cause of dementia and is characterized by a persistent deterioration of higher mental functions that results in an alteration of the individual’s ability to carry out activities of daily life [4]. It is the chronic disease that causes the most dependence, with about 90% of patients in a situation of high dependence, and 80% of them cared for by their families [4]. Severe mental disorders (SMDs) such as schizophrenia, bipolar disorders, and others, characterized by the severity of symptoms, long duration, and impairment of the person’s functionality at different levels, are a major health issue worldwide because of their high prevalence, impact, and disruption [7]. Many people affected live with severe and limiting disorder symptoms and sequelae, which affect their functionality and require support from professional or family caregivers [7]. SMDs encompass diagnoses on the schizophrenia spectrum, bipolar disorder, and some forms of depression [7].

Previous studies have shown that caregivers of older adults with neuropsychiatric disorders experience increased levels of anxiety and depression, as well as poorer quality of life [4,5], in addition to facing social and economic constraints [5,7]. Likewise, when specifically studying the unmet needs of caregivers of people with dementia, Zwingmann et al. [8] concluded that despite 24.3% of them not having any, most had a wide variety of unmet needs, with the most common being caregiver support groups, caregivers’ quality of life and mental health, and coping with depression and anxiety [8].

The current evidence suggests a need for comprehensive policies to address the needs of family caregivers of people with CCs [1]. To that end, the long-term impact of caregiving and the unmet needs of family caregivers must be determined to design, implement, and analyze strategies based on the person’s preferences and possibilities for successful self-care and caregiving experience in the family. However, to our knowledge, no previous studies have adopted a holistic perspective to address the impact, the unmet needs, and the developed self-care strategies for the achievement of successful self-care and long-term caregiving experiences in family caregivers of people with neuropsychiatric disorders based on their preferences and possibilities.

## 2. Materials and Methods

### 2.1. Research Questions, Aims, and Objectives

Our research questions were as follows: what is the impact of long-term caregiving on experienced family caregivers? Which unmet needs do they present due to the impact of long-term caregiving? Which self-care strategies could contribute or otherwise to a successful long-term caregiving experience?

The aims of this study were to identify the impact of caring in long-term family caregivers and the unmet needs with a holistic approach and to explore the self-care strategies developed by family caregivers to achieve or not a successful long-term caregiving experience.

The specific objectives of the study were as follows:

- To describe the sociodemographic characteristics and clinical data of family caregivers and caregivers’ recipients.
- To explore the health situation of family caregivers with a holistic approach, including caregiver burden, anxiety or depressive symptoms, sleep quality, quality of life, self-care, adherence to the Mediterranean diet, self-reported physical activity, and body mass index.
- To understand the impact of long-term caregiving and the unmet needs from the family caregivers’ perspective.
- To identify the self-care strategies developed by family caregivers.

### 2.2. Design of the Study

A mixed-method approach was used through semi-structured interviews and a questionnaire in which standardized, self-reported measures of burden, health behaviors, sleep, and mental well-being were administered. Mixed-methods methodology provides a better understanding of the phenomenon studied from different perspectives, comparing findings obtained from the interviews and questionnaires or scales [9].

### 2.3. Study Setting and Sampling

The participants were family caregivers of persons with a severe mental disorder (SMD) or with Alzheimer’s disease (AD) living in Valencia, Spain. The context was two non-governmental organizations: *Asociación para la Salud Integral del Enfermo Mental* (ASIEM, which means Association for the Integral Health for the Person with a Mental Health Disorder in Spanish) and *Asociación de Familiares de Enfermos de Alzhéimer* (AFAV, which means Association of Relatives of Persons with Alzheimer’s Disease in Spanish). ASIEM, founded in 1999, has more than 900 members and works with people with a psychiatric diagnosis and their families. AVAF, founded in 1991, helps people with AD and their families to achieve a better quality of life. It has over 2500 members and provides out-patient care for over 8000 people.

### 2.4. Inclusion and Exclusion Criteria

Being a family member and primary caregiver of a person with AD or an SMD for at least three years (if this responsibility was shared with another person, they could both participate in the study); the ability to speak and understand Spanish; willingness to participate. Those with a cognitive impairment that limited their participation in the interview were excluded.

A convenience sampling procedure was used to recruit the participants. The authors contacted the organizations and requested permission to present the study to potential participants. Then, an information meeting was organized in both organizations to introduce the research group, explain the purpose of the study, and invite those present to participate. To avoid potential volunteers feeling pressured to participate, in one of the organizations, ASIEM, a notebook was left in the meeting room so that volunteers could provide their names and contact details for the research team to arrange an interview. In AFAV, those caregivers willing to participate gave their contact details to the nurse of the organization, and the researchers were given a list of potential participants. Two of the researchers (SC and VSM) contacted volunteers, checked whether potential participants met the inclusion criteria, and explained the study; they all agreed to participate. Not all volunteers were interviewed as data saturation was achieved. A total of 28 family caregivers were included in the study: 13 cared for a person with a mental health disorder, and 15 cared for a person with AD.

### 2.5. Data Collection

#### Qualitative Methodology

Semi-structured interviews were used to explore the impact and unmet needs of family caregiving of people with SMD and AD. This study was reported following the Consolidated Criteria for Reporting Qualitative Research, COREQ [10,11,12]. One of the researchers conducted the interviews with the participants face-to-face. These researchers had training in qualitative research through master’s or doctoral degree courses and previous experience undertaking qualitative studies through interviews and focus groups. Each interview was scheduled at a suitable time and location for the volunteers. They were invited to choose the location for the meeting to make them feel comfortable and to facilitate cooperation. Eleven interviews occurred in the participants’ homes, eight in the organizations’ offices, and nine at the Faculty of Nursing and Podiatry. Only the interviewee and the interviewers were present to allow the interviewee to express themselves freely. The observer took notes on the participants’ non-verbal expressions to obtain more comprehensive information. None of the participants had any prior relationship with the researchers.

The researchers explained the study in detail to the participants before starting each interview. They were then invited to sign the informed consent and to give their permission to the conversation being recorded. All volunteers signed the consent form and agreed to audio recording the conversation. The semi-structured interviews followed a script to ensure the research objectives were met. The first question aimed to determine each participant’s context and caregiving situation, while the others focused on the specific objectives of the study. The researchers took field notes during the interview. The interviews lasted between 45 to 90 min. The sample size was determined when data saturation was reached [10]. It was achieved in this study with 26 interviews, and 2 more participants were interviewed to confirm it.

### 2.6. Data Analysis

The recordings were transcribed verbatim, including the notes taken during the interviews. The observer performed the transcriptions. For the content analysis, the three authors pre-analyzed the texts through several readings of the primary documents to create the first list of topics. The quotations were generated, and the most relevant text segments were selected and coded. Then, relationships were established, and code categories and themes were created to infer the phenomenon from the informants’ discourses. Finally, the correspondence between the transformed and the original data was verified. The coding was undertaken inductively and deductively. Several a priori topics were defined following the conceptual framework, while other topics or codes emerged as the analysis was conducted. The content analysis included a triangulation of researchers to increase the rigor of the analyses, and as such, each interview was analyzed by two researchers. For the correct interpretation of data, when consensus was not achieved, the third researcher reviewed the transcription and participated in the decision. Atlas.Ti (version 8) was used for the analysis. This software package is a tool for content and discourse analysis of qualitative data that facilitates its management, organization, and interpretation [11].

### 2.7. Quantitative Methodology

#### Instruments with Validity and Reliability and Data Collection

Once the qualitative interview was finished, the quantitative data collection started. A form including sociodemographic and clinical data of the family caregivers and the person affected was completed, and several quantitative instruments were administered. These questionnaires measured caregiver burden, anxiety or depressive symptoms, sleep quality, quality of life, self-care agency, adherence to the Mediterranean diet, self-reported physical activity, and body mass index. Since questionnaires might invite the participants to express some issues that were not reflected in the interview, this part of the data collection was audio-recorded. The questionnaires were administered verbally, and the researcher recorded the responses.

The Zarit Caregiver Burden Interview was created by Zarit et al. (1980) and adapted and validated in Spanish [13]. This scale is designed to assess the burden of caregivers of persons with dementia. It comprises 22 items that assess the negative impact on specific areas of daily life associated with caregiving: physical health, psychological health, social activities, and economic resources. Unlike the original, the version validated in Spain [14] includes a 5-point Likert scale for a total score ranging from 22 to 110. In this study, different cut-off points are proposed: from 22 to 46, no burden; from 47 to 55, with burden; and from 56 to 110, intense burden. This scale has demonstrated adequate psychometric properties, good construct validity, and high reliability (Cronbach’s alpha = 0.91 and test-retest = 0.71) [14]. It is a scale that seems particularly suitable for measuring the burden of caregivers of older adults with any type of dementia [14], as is the case of this project.

The Anxiety and Depression Scale, developed by Goldberg et al. [15], is a screening instrument for adults and older adults with 18 items. It includes four screening questions for anxiety and four for depression. If there are two or more affirmative answers to the initial questions on the anxiety scale (one or more for the depression scale), five more questions should be asked. The final score is the number of affirmative answers for each subscale. Individuals with anxiety scores ≥ 4 or depression scores ≥ 2 are considered to have a 50% chance of clinically significant disturbance. This instrument was validated in Spanish by Montón et al. (1993) [16], with adequate sensitivity (83%) and specificity (81.1%) and a 95% positive predictive value.

The Athens Insomnia Scale is a screening tool for sleep disturbances, which uses a self-reported questionnaire. The full scale is based on the International Classification of Diseases (ICD-10). This scale includes eight items, and the scores range from 0 to 24 (with higher scores suggesting a more severe problem and a cut-off point of six). It was validated by Soldatos et al. in 2000 [17] and validated in Spanish by Gómez-Benito et al. [18] with acceptable psychometric properties (Cronbach’s alpha was 0.86) in 2011.

The Short Form-36 Health Survey (SF-36) is validated in Spanish [19]. It is a 36-item short-form (SF-36) constructed to survey health status in the Medical Outcomes Study. The SF-36 was designed for use in clinical practice and research, health policy evaluations, and general population surveys [19]. The SF-36 includes one multi-item scale that assesses eight health concepts: (1) limitations in physical activities because of health problems; (2) limitations in social activities because of physical or emotional problems; (3) limitations in usual role activities because of physical health problems; (4) bodily pain; (5) general mental health (psychological distress and well-being); (6) limitations in usual role activities because of emotional problems; (7) vitality (energy and fatigue); and (8) general health perceptions. The survey was constructed for self-administration by persons 14 years of age and older and for administration by a trained interviewer in person or by telephone. This instrument addresses health concepts from the person’s perspective. The SF-36 scores range from 0 to 100.

The Appraisal of Self-care Agency Scale (ASA-S) was measured based on a 24-item scale in which each item was scored on a 5-point Likert scale [20]. The total ranged from 24 to 120. Higher scores indicated a better capability to care for personal health and procure well-being. Since there are no accepted cut-off scores, we have divided the scores obtained in the sample into quartiles, and participants whose scores are included in the highest quartile will be classified as having good self-care capacity. The scale was originally designed in English and has been translated into several languages, including Spanish [21]. Studies have been carried out to determine the properties of the scale in the Spanish-speaking population, obtaining good psychometric results. A study carried out with an adult population over 65 years of age with chronic conditions demonstrated the conceptual validity and reliability of the scale, obtaining a Cronbach’s alpha value of 0.77, which means that the items of the scale measure the same concept (internal consistency) [21].

Since there are no accepted cut-off scores for the SF-36 and ASA scale, we divided the scores obtained in the sample into quartiles, and the participants whose scores were included in the highest quartile were considered as having good HRQL and self-care, respectively.

Adherence to the Mediterranean diet as a standard of a healthy diet was measured using a validated 14-item scale that obtained adequate validity and reliability properties [22], with higher scores indicating better adherence. An overall score of under 9 points represents low adherence, while an overall score of 9 points or more identifies participants with high adherence to the Mediterranean diet.

Self-reported physical activity data were collected using the short form of the International Physical Activity Questionnaire (IPAQ]). This is a 7-question instrument about the frequency, duration, and intensity of activity (moderate and intense) performed in the last seven days and walking and sitting time in a working day. Respondents are asked to report the time spent in the previous seven days in physical activity performed during leisure time, at work, domestic activities, and transport at three intensities: walking, moderate, and vigorous [23]. The participants were considered to meet the recommendations for physical activity if they reported at least 150 min per week of walking or moderate or vigorous physical activity. This scale had good reliability coefficients for its application in the Spanish population (Cronbach’s alpha of 0.82) [24].

Body-mass index (BMI) was calculated as the body mass divided by the square of the body height and is expressed in units of kg/m^2^, resulting from mass in kilograms (kg) and height in meters (m). BMI was further categorized according to the WHO criteria [25] as underweight (<18.5 kg/m^2^), normal (18.5 to 24.99 kg/m^2^), overweight (25.0 to 29.99 kg/m^2^), and obese (≥30 kg/m^2^). A BMI ≥ 25.0 kg/m^2^ denotes overweight and obesity.

### 2.8. Statistical Analysis

The quantitative variables were reported as mean values with the standard error mean, and the scores obtained were considered as under or over the cut-off scores of the instruments applied. The categorical variables were described as frequencies and proportions. Statistical analysis of the associations between quantitative variables and categorical data was performed using the non-parametric Mann–Whitney U test (supported by SPSS, the Statistical Package for Social Science software version 26.0).

### 2.9. Ethical Considerations

The Human Research Ethics Committee of the University of Valencia approved the research on 5 September 2019 (protocol number 45874). The confidentiality of the data was guaranteed. Permission to record the interviews was requested, and all the participants signed an informed consent form once they received all the information necessary about the details of the study.

## 3. Results

**Figure 1:**
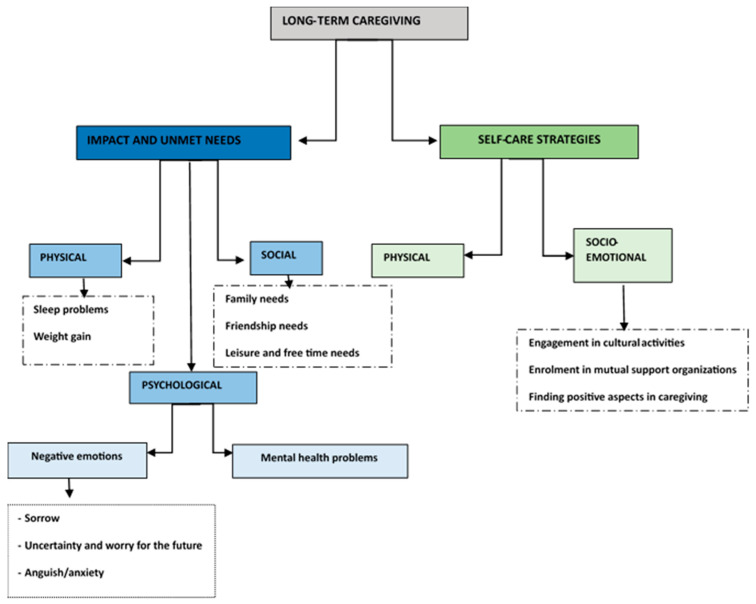
Themes, subthemes, and categories/codes.

As a mixed-method study, both qualitative and quantitative data were combined during the data analyses, and two main themes (with subthemes and categories/codes) were identified as a result: (1) the impact of long-term caregiving and unmet needs of caregivers and (2) caregivers’ successful self-care strategies (Figure 1).

### 3.1. Characteristics of the Sample

Twenty-eight caregivers participated in this study (Table 1), with a mean age of 69.0 ± 9.7 years; 18 (64.3%) were female, 24 (85.7%) were married, and 23 (82.1%) were retired. All the caregivers except one had chronic disorders; 23 caregivers had more than one chronic disorder, and 18 out of 28 were receiving pharmacological treatment for these chronic conditions. The participants’ and the care recipients’ characteristics are described in Table 1.

### 3.2. Impact of Long-Term Caregiving and Unmet Needs of Caregivers

This broad theme was organized into subthemes: physical, psychological, and social impact and unmet needs.

#### 3.2.1. Physical Impact and Unmet Needs

The participants said that caregiving had a physical impact on them. This finding was supported by the quantitative data, as 64% (n = 18) had a suboptimal health-related quality of life according to their SF-36 scores (Table 2). As a result, the participants presented unmet physical needs related to long-term caregiving, which were classified into four categories: sleep problems; weight gain; exhaustion; and reduced self-care.

#### 3.2.2. Sleep Problems

Several participants reported having sleep problems related to long-term caregiving. For example, some referred to poor quality sleep, while others said their hours of sleep were insufficient or they needed medication to sleep. The quantitative results confirmed this finding, as 36% (n = 10) reported poor quality sleep according to the scores obtained using the Athens Insomnia Scale (Table 2).

#### 3.2.3. Weight Gain

Some participants felt that long-term caregiving had increased their body weight due to various factors. For example, they mentioned the use of psychotropic drugs, not taking proper care of their diet, and a decline in their physical exercise. One of the participants described previously being overweight and returning to a normal weight after one year on a diet but subsequently becoming overweight again. In the quantitative results, eight caregivers (28.6%) presented an altered body mass index; four were overweight and four were obese (Table 2).

#### 3.2.4. Exhaustion

Other participants reported feeling mentally and physically exhausted by caregiving. This exhaustion impacted other areas of their self-care, leading them to feel little energy for doing physical exercise or eating healthily.

#### 3.2.5. Reduced Self-Care

As reflected by the ASA-S scores, 71% of the sample (n = 20) presented suboptimal self-care (Table 2). Several family caregivers considered their self-care limited and said they were conscious of neglecting their health, exercise, or diet. Several caregivers stated this was due to exhaustion, while others mentioned a lack of energy or time. These findings are also supported by the quantitative data, which, in addition to a significant percentage of overweight and obesity, indicated suboptimal adherence to the Mediterranean diet of 21% (n = 6). Moreover, 50% (n = 14) had low levels of physical activity measured using the IPAQ (Table 2).

C1: (…) I don’t go to the doctors, I don’t go anywhere because I don’t want to or feel like it, and when I can, I lie down on the couch to sleep (…) because you’re tired out, it’s exhausting.

### 3.3. Psychological Impact and Unmet Needs

The participants reported various perceived psychological needs. Three categories were identified: negative emotions, emotional discomfort, and mental health problems.

#### 3.3.1. Negative Emotions and Emotional Discomfort

Some caregivers expressed a range of negative emotions and emotional discomfort. These included sorrow, uncertainty and worry about the future, anguish/anxiety, loss of the ability to disconnect, and guilt.

#### 3.3.2. Sorrow

Long-term family caregivers expressed sorrow for the person they cared for. However, the caregivers of people with dementia more frequently expressed sorrow related to the progression of the illness and the patient’s deterioration and need for more care.

Some caregivers expressed suffering and sorrow about the pain they saw the relative they cared for experiencing.

#### 3.3.3. Uncertainty and Worry About the Future

Several caregivers said they were worried about the future of the person they cared for, both in terms of the evolution of their illness and the possibility that they might not be there to help them in the future. In particular, the caregivers of people with dementia said they felt fear when they thought about the possibility of having health problems that prevented them from caring for their loved ones and even about the possibility of dying before the ill person. In some cases, their main worry was that the illness would progress and the affected person would need more care:

C10: “I can’t get over my husband’s situation; I can see how he’s getting worse every day”.

Several caregivers indicated that they saw the future as entirely black, pessimistic, and fearful.

#### 3.3.4. Anguish/Anxiety

The caregivers said long-term caregiving was a source of stress because it was difficult to care for the person, and they felt a huge burden. Some caregivers said they had experienced extreme situations due to their relative’s illness. One of the participants said that her mother had become very aggressive and that it was complicated to deal with her, which made her desperate:

C7: “She’s managed to bring out the worst in me; I’ve even wished she would die…”

Other caregivers expressed feeling desperate about being unable to help the relatives they cared for to solve their problems.

#### 3.3.5. Loss of the Ability to Disconnect

Some caregivers said they felt constantly engaged in caring or paying attention to the person they cared for, limiting their ability to focus on other things.

C13: “And that’s it, that’s my life, always keeping an eye on him”.

#### 3.3.6. Guilt

Several participants expressed feelings of guilt. Some blamed themselves for previous situations in which their relative’s condition had deteriorated. In contrast, others did so by taking some time off caring while they left the person in care.

#### 3.3.7. Mental Health Problems

Several caregivers said that they had been diagnosed with mental health problems related to caregiving, which were mostly anxiety or depression. These findings were also apparent in the quantitative results. Based on the cut-off scores, 32% (n = 9) of the caregivers had a high caregiver burden, 43% had a high level of anxiety symptoms, and 36% (n = 10) had high levels of depressive symptoms as reflected by the scores obtained in the Anxiety and Depression Goldberg Scale (Table 2).

### 3.4. Social Impact and Unmet Needs

According to the scores obtained in the Zarit Questionnaire, 32% of the sample felt they had a high caregiver burden (Table 2). In the interviews, the caregivers referred to important unmet social needs due to the impact of long-term care. These social needs can be categorized as family, friendship, leisure, free time, and economic and household needs.

#### 3.4.1. Family Needs

Support from the family was one of the main factors identified in meeting the caregivers’ needs. In most cases, their family members were vital for the caregivers, and they stated this unambiguously. Only one caregiver said the situation helped them strengthen their family ties.

Some caregivers said that although their children were mainly giving them emotional support, they were unable to care for the ill person because they had their responsibilities and families. Other caregivers of people with SMD made excuses for their offspring and said that caring for their children was only the parents’ responsibility and wanted to protect and respect their other children:

C25: “My daughters and their husbands need to get on with their lives (…) I’m all alone, but don’t ever tell me that I made a mess of this. I think that it is what it is. In other words, their father and I have the problem (…). These things are for me to deal with. I’ll make my bed, and I’ll lie in it”.

Many caregivers felt that they were neither understood nor supported by their families. In some cases, the family even believed that the caregivers were to blame for the situation:

C18: “One of my children doesn’t understand the situation, and he simply says that it’s our fault; why don’t we put him (i.e., the affected person) out on the street to work”.

For some caregivers, caring for the person with the disease was seen as a significant sacrifice because their life was entirely devoted to the other person. They stated that their total involvement with the person negatively affected their relationship with their other relatives, including their children, and their relationship with their siblings. In several cases, while the mother was fully engaged in caring for her son, the father did not accept the situation, and the relationship between them was very tense or had broken down.

C19: “We’ve been living in the same house for two years, but we don’t speak to each other, (…) we don’t live together; we don’t eat together; He sleeps in the big bedroom, and I sleep in another one”.

Some caregivers stated that the most challenging situation for them was that they no longer had any privacy with their partner since they had been living with the person receiving care:

C28: “We live with someone who is always watching us, and we can’t talk about anything. What I miss most is the privacy my wife and I could have regarding being a couple”.

Several caregivers experienced loneliness. For instance, the caregiver of a woman diagnosed with AD said that he felt alone as his wife was at the center all day, and they were no longer able to do things together:

C6: “I’m on my own all day; it’s as if I was a widower”.

#### 3.4.2. Friendship Needs

Most caregivers indicated that they had friends, which was relevant to them. Having free time was, therefore, clearly a determining factor in meeting caregivers’ needs regarding friendship. However, they often mentioned that they needed time with their friends. In some cases, the impact of caring left them without time to meet other people. As a result, some said that apart from their family, they had lost their friends. On the other hand, very few caregivers noted that they did not meet their friends because they did not want to go out with them anymore.

C11: “My daughter encourages me to go to the pensioner’s center, but I don’t feel like it. I’ve never been one for bars. I prefer to stay home”.

#### 3.4.3. Leisure and Free Time Needs/Respite Time

Caring for their relative was often so demanding that the caregivers said that they did not have any time for themselves, and they felt that their lives were passing them by:

C1: “They depend on you so much that you have no life of your own”.

In addition to caring for the ill person, some caregivers also had to care for their grandchildren:

C8: “My other daughters don’t help me. I don’t have time for anything. And what’s more, I take care of their children, my grandchildren”.

In some cases, they reported burnout due to the impossibility of making plans and having free time. Some caregivers also said they missed the leisure time they shared with the person with the disease, i.e., going on holiday together. Traveling was one of the most important leisure activities for the caregivers. Some of them still went on holiday, but others had to give it up. One of the reasons for this was that their relative could no longer deal with the situation.

Another significant aspect that some caregivers had to give up was going to their second home at weekends and during summer. Other caregivers said that they had had to give up their hobbies entirely.

#### 3.4.4. Economic and Household Needs

The caregivers reported facing economic constraints due to their situation with the person with the disease. These financial constraints limited the caregivers in several ways, particularly the days the affected person could spend at the day center. The caregivers also said they would need help at home for cleaning and the patient, but they did not have enough money for this.

Several caregivers caring for their son or daughter reported a significant financial impact due to the illness; in one case, they even needed to buy a new house:

C28: “We have changed our lives. His mother and I sold the apartment we had, and we moved to a bigger one because, at his age, it’s tough for him (the affected person) to have financial stability, so he has to live with us”.

Caregivers of people with SMD said they had many arguments related to money. Their son had addiction issues (gambling disorder), and they needed increasing amounts of money. Moreover, caregivers added the financial impact of paying lawyers to settle debts and deal with other financial problems created by their affected relatives.

Caregivers also mentioned the need to access welfare policies to obtain financial support. Some were already receiving financial support, while others were waiting for it.

### 3.5. Caregivers’ Sense of Abandonment by Professionals

Some participants said they felt the professionals had not provided enough information, help, or guidance to manage their caregiving.

C23: “They could provide courses for caregivers on how to manage anxiety or exercises to deal with stress because anxiety is what affects us the most, as well as the diseases that come from anxiety and somatization”.

### 3.6. Caregivers’ Successful Self-Care Strategies

The caregivers said they were aware that they had to care for themselves, and most of them could apply one or more strategies. These self-care strategies involved varied attitudes, habits, or actions: physical and socioemotional self-care strategies.

### 3.7. Physical Self-Care Strategies

Most caregivers recognized the need to maintain healthy lifestyle habits to care for themselves. These habits were mainly focused on physical care and consisted of physical activity, a healthy diet, and wellness practice.

The physical activity described by family caregivers was varied. Some caregivers said they did guided exercise at home, walking or playing sports, either alone or in groups. For caregivers, this physical activity not only assisted them with their physical health but was also a source of social relations and emotional well-being.

Another healthy habit described by participants was adherence to a healthy diet adapted to their health. Other participants reported doing wellness activities, such as yoga and Tai Chi.

### 3.8. Socio-Emotional Self-Care Strategies

#### 3.8.1. Engagement in Cultural Activities

Some participants enrolled in cultural activities, such as attending lessons at university, reading, listening to music, or painting. These activities not only helped them to relax but, in many cases, also led to the creation of new social networks.

#### 3.8.2. Enrolment in Mutual Support Organizations

All caregivers stressed that the organization had been helpful to them. In addition to providing them with professional support or advice, it also allowed them to rest and have free time. In addition, one of the aspects that they considered most helpful was attending the meetings for family members. In this context, they could share their feelings and experiences with individuals experiencing the same situations. In some cases, this resignation turned into consolation or comfort when they shared their caregiving experiences with other caregivers in mutual support groups, and they felt supported and understood.

Mutual support organizations also reinforced the caregivers’ anticipation of their relatives’ future. The organizations advised them on how to solve issues related to inheritance, inclusive regulations, and social welfare policies. The participants stated that they felt it was essential to believe that the situation would be under control and clear when they were no longer able to provide care or in the event of their deaths, which contributed to their well-being.

C24: “They told me about the options for support my daughter could receive. That was a relief then”.

#### 3.8.3. Finding Positive Aspects in Caregiving

Some caregivers expressed they had been able to turn towards the positive understanding that caregiving had helped them feel the satisfaction of being able to care, feel they were closer to the relative they cared for, and value important things.

#### 3.8.4. Respite Time

Long-term family caregivers referred to the need for leisure and free time for themselves to be able to develop the activities they enjoyed. It could be termed “respite time” alone.

C1: “I need to get away at times. To be free. Going out for a walk, shopping, or sunbathing does me good”.

Several participants said they felt supported in caregiving by their families, and these tried to free them from their responsibility to give them some respite. Maintaining a job had been helpful for other caregivers. This kept their mind busy, and they met people and friends, which immensely helped them.

In order to facilitate the description of the codes, subthemes, and themes identified in qualitative data, only some of the most representative citations have been maintained in the manuscript; the other citations can be consulted in Table 3.

### 3.9. Analysis of Caregiver Characteristics

As reflected in Table 4, there were no statistically significant differences between caregivers of people with SMD and those with AD in terms of age, sleep quality, anxiety symptoms, depressive symptoms, adherence to the Mediterranean diet, body-mass index, and self-care agency. In contrast, caregivers of people with AD spent significantly less time engaging in physical activity, had higher caregiver burden, and poorer health-related quality of life.

## 4. Discussion

This is one of the first studies that has adopted a mixed-methods perspective to address the impact, unmet needs, and self-care strategies for achieving a successful long-term caregiving experience among family caregivers of people with neuropsychiatric disorders. One of the primary and most novel findings of this study is that some caregivers presented successful self-care strategies, which cannot be considered purely physical, emotional, or social self-care activities, but they may be cross-sectional or at least most of them have a psychological–emotional component.

The sample was composed of family caregivers of people with neuropsychiatric disorders. This study suggests that the specific neuropsychiatric disorder affecting the person is not the main determining factor in the caregiving experience. We confirmed this hypothesis as no significant differences in most explored outcomes between AD and MH caregivers were found.

We found no differences in family caregivers’ burden according to their years of caring. Instead, the caregivers’ burden was mainly associated with their psychological characteristics, and it was correlated with depressive and anxiety symptoms, poor sleep quality, and health-related quality of life. This finding suggests that even under these long-term conditions, the mental health of caregivers is at least partially related to the burden [26] because the number of years spent caregiving and the age of caregivers were not associated with the burden, as in other studies [27]. The ability to self-care assessed by a self-care agency was also significantly associated with the caregivers’ burden, suggesting that caregiving with a greater burden leads to a reduced engagement in activities to maintain adequate self-care. Research on self-care among informal caregivers of people with neuropsychiatric disorders is scarce and has recently been undertaken in caregivers of people with autism and schizophrenia [28]. This association does not appear to be specific to the characteristics of the care recipient since it has been reported in various studies of caregivers of patients with cancer, in which the subjective burden was negatively and significantly related to self-care agency [29].

When analyzing self-care practices, we observed that half of the caregivers had low physical activity levels compared to those recommended by WHO [30], and one-third had a highly sedentary lifestyle with practically no physical activity. As regards a healthy diet expressed as adherence to the Mediterranean diet, we observed that 21.4% of individuals had low adherence, with potentially harmful consequences related to cardiovascular and other diseases. Obesity was present in 18% of the participants (with no explicit parallelism to poor adherence to the Mediterranean diet), and one person was underweight, suggesting nutritional patterns could be one of the aspects for consideration when dealing with caregivers in individualized interventions aimed at improving self-care abilities. However, the caregiver’s burden level was not significantly associated with weekly physical activity, and BMI was not associated with adherence to the Mediterranean diet. Greater social isolation among older men and women has been related to reduced everyday objective physical activity and increased sedentary time [31]. Differences in physical activity may contribute to an increased risk of ill-health and poor well-being associated with loneliness and poor social support [32].

The impact and needs of long-term caregivers regarding self-care patterns differ [33]. Some caregivers develop a successful pattern in which they can use strategies and engage in activities that help them achieve successful self-care and caring experiences. A systematic review by Lindeza et al. (2020) [34] concluded that some conditions, such as the support provided by appropriate formal care and family and friends, helped caregivers of people with dementia to obtain a positive caregiving experience. In our study, the caregivers described feeling abandoned by the health system, but they looked for support from organizations.

Many individuals with a high measured burden showed poor self-care and quality of life due to long-term caring, which had a considerable impact on their lives, generating needs at various levels: physical, psychological, and social. This finding is consistent with previous research, which concluded that caregiving to people affected with AD negatively influenced the caregivers’ self-care management, including physical self-care and mental and social self-care [35]. Likewise, Waligora et al. (2019) [36] concluded that the self-care needs of caregivers of people with dementia included sleep, social engagement and support, and leisure activities. These authors also identified various barriers to self-care: gender roles, self-sacrificing, minority ethnicity, and the burden of caregiving.

At the physical level, the caregivers presented sleep problems, weight gain, and reduced self-care, among others. They also experienced emotional suffering due to the long-term care. The caregivers reported feelings of sorrow, anxiety, guilt, and loss of the ability to switch off. In a study carried out in Greece in 2012, the distress experienced by caregivers of people diagnosed with schizophrenia was analyzed. It concluded that it was related to the caregivers’ female gender and specific clinical features of schizophrenia: the years since the diagnosis and the severity of the symptoms [37]. However, as reported by Rhee and Rosenheck (2019) [38], small clinical changes in adults with schizophrenia might not be enough to affect long-established caregivers’ experience of the burden.

At the social level, caregivers mainly felt an impact on their family and friendship relationships, and they needed leisure time and respite. Moreover, they mentioned some professional and economic implications due to caregiving. These findings are consistent with previous research, which indicates that family caregivers are at an increased risk of suffering physically, psychologically, and socially while providing care for family members with mental health conditions [39]. More specifically, Attep Özden (2018) [40] stressed that caregiving for people with schizophrenia affects the caregivers’ quality of life and their social and financial situation. The caregivers in the present study did not identify any spiritual needs. However, previous research has found that being a caregiver of a person with a neuropsychiatric disorder impacts a person’s spirituality, and caregivers expressed spiritual needs, such as hope and finding meaning in life and spiritual or religious practices that help them cope with stressful situations [41]. Similarly, other authors indicated that the adverse effects of caregiving can be offset by social support and religious or spiritual beliefs [33].

The long-term experience of caregiving did not prevent some of the participants in our study from improving their experience and well-being by developing strategies based on their interests, preferences, and real possibilities. These strategies for long-term caregiving have also been mentioned in the literature. A recently published scoping review [33] suggested that some family caregivers can become more resilient over time, grow into stronger, more adaptable, and healthier people [42], and improve the relationship with the person they care for while also providing a sense of inner strength and satisfaction [43].

In their study, Pope et al. (2017) reported that caregivers’ self-care was inversely related to their perceived stress and pain and directly associated with general health and emotional well-being [44]. This implies that caring for the caregivers involves assessing these factors and providing them with effective strategies to reduce stress and enhance their general health and emotional well-being. According to Waligora et al. (2019), promoting self-care among caregivers also requires acknowledging the personal consequences of caregiving, balancing self-care needs with the affected person’s needs, and positioning oneself as a duality, separating their personal from their caregiving roles [36].

This study has stressed the importance of individualized care. Assessing and understanding the individual’s preferences is necessary to design tailored and adequate strategies that may improve their quality of life and caring experience. In a recent study, this idea was also stressed, suggesting that future interventions to promote family caregivers’ well-being may need to consider personality, particular circumstances, and cultural and personal beliefs [33]. In addition, ascertaining the environment in which the caring experience takes place is crucial, e.g., the degree of family support provided, to adapt the strategies to the real possibilities.

Some strategies have multiple and cross-sectional benefits for caregivers. For instance, physical activity such as walking need not only to be physical, but it could also mean contact with nature and produce positive feelings, thereby having a positive psychological impact, or also improving the caregivers’ social dimension if the activity is shared with other people. Knowledge of the caregiver’s interests could help care planning to improve their physical, social, emotional, and spiritual dimensions.

This study presents strengths and limitations. Its strengths include its description of the long-term caring experience and strategies for self-care from the caregivers’ perspective. A potential limitation of this study could be that all the family caregivers belonged to patients’ organizations, which offer them practical information and mutual support. Moreover, all of them lived in urban areas. These aspects could have influenced the results, and it would be interesting to carry out future studies comparing different populations. The qualitative methodology has important limitations that should be taken into account. In any semi-structured interview, the interaction between interviewer and interviewee can affect the quality of the interview. In describing their situation, we may have had a response bias such as social desirability, and participants may have consciously or unconsciously tried to respond as they thought the interviewer expected. Our impression was that participants openly shared their experiences and perceptions of caregiving-related factors. However, we can assume that those who refused to be interviewed probably found it more challenging to communicate their situation than those who consented. Critical reflection and discussion in the research group throughout the process, especially in the systematic analysis of the interviews, helped to create the necessary distance to capture the essence of the participants’ communication.

As a limitation in this study, to quantify self-reported measures of burden, health behaviors, sleep, and mental well-being, we used the proposed cut-off for many of the scales used in this study and dichotomized self-care agency and quality of life to arbitrarily group divided into high or low scores. Such categorization results in lost information, reduced power of statistical tests, and increased probability of a Type II error [45].

Our recommendations for further research comprise studying the phenomenon, including a more heterogeneous population in terms of the caregivers’ characteristics, looking at possible differences between living in rural and urban areas, receiving support from organizations or not, having comorbidities, gender differences, and family relationships.

## 5. Conclusions

Family caregiving for people with neuropsychiatric disorders was found to affect various areas based on participants’ reported impacts. The physical impact of caregiving is manifested by poor sleep quality, weight gain, reduced self-care, and exhaustion in the many caregivers in our study. The psychological impact is related to emotional discomfort and mental health problems. Caregiving may cause unmet social needs in family and friendship relations, a lack of time for the caregiver, and economic difficulties, combined with a sense of abandonment by professionals.

In contrast, some family caregivers experience low burden levels and describe successful self-care strategies that are physical and socio-emotional. Physical self-care comprises healthy habits and physical activity, while socio-emotional self-care implies engaging in cultural activities and with mutual support organizations, being able to find positive aspects in caregiving, and having respite time. Helping caregivers to prevent or manage the negative impact of caregiving implies an assessment of their circumstances, strengths, and interests in order to develop cross-sectional self-care strategies involving the physical, emotional, and social spheres.

## Figures and Tables

**Table 1:** Caregivers’ and care recipients’ characteristics.

Caregivers’ Characteristics							Care Recipients’ Characteristics		
Alias	Age	Gender	Years Caring	Number of Chronic Conditions	Kinship	Help at Home	Disorder	Age	Gender
C1	53	F	10	1	Daughter	Yes	AD	86	F
C2	82	M	3	1	Husband	Yes	AD	80	F
C3	73	M	20	2	Husband	No	AD	75	F
C4	72	M	4	1	Husband	No	AD	75	F
C5	83	F	3	1	Wife	No	AD	84	M
C6	77	M	11	1	Husband	Yes	AD	78	F
C7	57	F	13	1	Daughter	Yes	AD	84	F
C8	66	F	3	2	Wife	No	AD	68	M
C9	80	M	6	1	Husband	Yes	AD	79	F
C10	80	F	8	1	Wife	No	AD	80	M
C11	76	M	3	2	Husband	No	AD	77	F
C12	53	F	3	1	Wife	No	AD	56	M
C13	66	F	12	2	Wife	No	AD	75	M
C14	84	M	15	1	Husband	Yes	AD	87	F
C15	69	F	14	1	Wife	No	AD	68	M
C16	61	M	15	3	Father	Yes	SMD	34	F
C17	63	F	20	1	Sister	Yes	SMD	50	M
C18	70	F	20	3	Mother	No	SMD	33	M
C19	70	F	6	2	Mother	No	SMD	23	M
C20	75	F	25	2	Mother	Yes	SMD	47	M
C21	79	F	24	1	Mother	No	SMD	44	M
C22	49	F	7	1	Mother	No	SMD	21	M
C23	59	F	10	2	Sister	Yes	SMD	61	F
C24	74	M	10	2	Father	No	SMD	45	F
C25	71	F	10	2	Mother	No	SMD	40	M
C26	65	F	14	3	Mother	No	SMD	36	F
C27	58	F	3	2	Mother	Yes	SMD	40	M
C28	59	M	3	0	Father	Yes	SMD	40	M

**Table 2:** Family caregivers’ situation according to the scores obtained in different scales: caregiver burden, depressive and anxiety symptoms, sleep quality, health-related quality of life, body mass index, adherence to the Mediterranean diet as a gold standard pattern in Spain, and amount of weekly physical activity by participants as described in the Methods section. Green boxes mean scores within average values, while red boxes mean altered values based on published psychometric cut-off values for each scale.

Alias	Caregiver Burden	Anxiety Symptoms	Depressive Symptoms	Sleep Quality	Self-Care Agency	Quality of Life	Body-Mass Index	Adherence to Mediterranean Diet	Physical Activity
C1									
C2									
C3									
C4									
C5									
C6									
C7									
C8									
C9									
C10									
C11									
C12									
C13									
C14									
C15									
C16									
C17									
C18									
C19									
C20									
C21									
C22									
C23									
C24									
C25									
C26									
C27									
C28									

**Table 3:** Citations by category.

Theme	Subtheme	Category	Citations
**IMPACT OF LONG-TERM CAREGIVING AND UNMET NEEDS**	PHYSICAL IMPACT AND UNMET NEEDS	**Sleep problems**	*C10: I take hypnotic drugs to be able to sleep; otherwise, I wouldn’t sleep… he’s in bed, and when he moves, it’s like an earthquake…* *C4: “[…] my head’s going over things all night […] on and on, all night …”*
		**Weight gain**	*C8: “I’ve put on a lot of weight, a lot; I don’t even want to weigh myself”.*
		**Reduced self-care**	*C27: “I often don’t feel like taking care of myself, eating healthily, for example”.*
	PSYCHOLOGICAL IMPACT AND UNMET NEEDS	**Negative emotions**	Sorrow *C25: “I’m very sorry about what’s happening to him”.* *C23: “It upsets me; it hurts me a lot because she has a terrible time. When she’s unwell, I get very upset”.* Uncertainty and worry about the future *C11: “I’m scared to think about what will happen when she gets worse”.* *C2: “If something happens to me, I don’t know what will become of her”.* Anguish/anxiety *C1: “I argue with her all day, which makes me stressed and upsets me a lot”.* *C13: “Sometimes I can’t do it anymore, and I say I’ve had enough”.* *C26: “It’s anxiety every day for me, an internal worry and pain, knowing that you have someone who isn’t well and who lives on his own. It’s a constant struggle. No matter how much the psychologist tells me that I have to live my own life, I can’t because he is my life”.* Guilt *C17: “Every time I go on holiday, I have a guilty conscience. I used to live somewhere else, and it happened to me then as well; it’s the worst thing I have to deal with”.* *C28: “I thought that giving him (the affected person) responsibilities could be the answer, but it made it much worse; he was even possibly very affected by the fact that I didn’t understand the situation […] His mother and I have blamed ourselves; we must have done something wrong”.*
		**Mental health problems**	C27: *“The doctor increased my antidepressant at my last appointment.”.* C13: *“I only get out of bed because my daughters and grandchildren need me […] I feel like I’m suffocating”.*
	SOCIAL IMPACT AND UNMET NEEDS	**Family needs**	*C20: “If my son weren’t ill, I would have been able to spend more time with my grandchildren. My daughter didn’t want to bring my granddaughter to see me if my son was here. If he wasn’t well, I had to stay with him. They say, come and stay for the weekend, mum, and I ask, “But how am I going to go to your house if your brother is here?”* *C1: “I’m my mother’s mother” […], “and it’s complicated with my daughter, very difficult because she hasn’t accepted that her grandma has this illness… and you can’t teach her… […] you can tell her a thousand times, write it down for her on a piece of paper, put a clock on it… it’s all the same. But it’s hard to understand that; it’s hard…”*
		**Friendship needs**	*C6: “Although I can’t always make it, I still try my best to have lunch with my friends”.* *C3: “I’ve lost everyone, my friends and even my family […] My relationship with my friends has completely deteriorated”.*
		**Leisure and free time needs**	*C3: “This isn’t a life or anything; I can’t do anything. My life is boring”.* *C25: “I haven’t done anything of mine these last few months. In other words, I prepare the food, do the dusting, sit down, and pretend to be enthusiastic, and that’s it. But I feel very sorry because I paint icons, and I’ve completely given that up because an icon requires affection, love… and a lot of peace to do it…”* C16: *“No leisure, no plans, no weekends, nothing. I’ve been like this for quite a few months without being able to do anything else. Completely paralyzed, just overcome by the problem”.*
		**Economic and household needs**	*C5: “I need a girl to come in and help me, but I don’t have the money for it”.* *C1: “Everything’s very expensive. I’ve had to buy a bed, a walker […]”* *C15: “He goes to the day center at the association, which is very helpful, but we’d like him to go for more days, and we can’t do it because it’s costly”.*
		**Sense of abandonment by professionals**	*C26: “With the mental disorder, we don’t have any help from anyone. She has an appointment with her psychiatrist every 4 or 5 months; she goes on her own. I think she needs an appointment with everyone who treats her, and together, we can get her out of her hole. The fact that they treat her with appointments so far into the future and that they don’t take her family into account makes me feel that the system is failing us”.*
**CAREGIVERS’ SUCCESSFUL SELF-CARE STRATEGIES**	PHYSICAL SELF-CARE STRATEGIES	**Physical self-care strategies**	C20: *“What relaxes me is going to the pool; I do 1.5 h of exercise, and then I get into the jacuzzi, and you interact with other people and switch off”.* C23: *When I get up in the morning, I do my exercise board guided by the physio for half an hour. I also go to pilates two days a week and therapeutic swimming one day; I exercise daily.*
	SOCIOEMOTIONAL SELF-CARE STRATEGIES	**Engagement in cultural activities**	C6: *“The others, it’s an enjoyable event because the people who are there are all quite nice, and so you spend time there, you paint, you look at the others, you get ideas, they give you ideas, whether you paint well or paint badly, You do your best, well, you know”.* *C20: “I go to class and university with many friends. I like to read; I’m in a book club. […]. I squeeze 24 h out of a day. I read, I go to class…”*
		**Finding positive aspects in caregiving**	C16: *“Really, what would make me happy now is having my son back because I got along very well with him when he was a child and a youngster, but we argued a lot starting when he became an adolescent.”.* C1: *“It’s a comfort to know that you’re taking care of her”.* *C17: “For me, it’s a satisfaction to be able to do it because I’m retired and have time”.*
		**Respite time time**	*C14: “I relax by writing and painting pictures”.* *C13: “My daughter always encourages me to go out and dance. I have a lot of friends there. We laugh, we don’t talk about anything important, and we switch off”.* *C12: “I need to work. Not only for the money but also to switch it off. It is the only place where I can do it”.*
		**Mutual support organizations**	C27: *“We found out about ASIEM, and we’re pleased because it’s made us think about all this a lot”.* *C1: “Sharing experiences with people who are going through the same thing is a comfort”.* C23: *“The organization has been a significant help because of the talks by the professionals who come to help you learn more about the disease, knowing that you have help there when you need it. With the group, knowing that you have their help and cooperation, and sharing many things that happen with the disease… They give you tools so you know how to cope with it. It’s been priceless”.*

**Table 4:** Comparison of quantitative variables between participant groups.

Self-Reported Measures of Burden, Health Behaviors, Sleep, and Mental Well-Being	MH (Mean ± Standard Deviation)	AD (Mean ± Standard Deviation)	*p*-Value
Age (years)	65.9 ± 8.4	72.7 ± 10.6	0.118
Duration of caregiving (years)	13.4 ± 7.6	8.0 ± 6.0	0.142
Caregiver burden (Zarit scale score score)	24.3 ± 14.2	41.8 ± 9.9	0.001 *
Anxiety symptoms (Goldberg scale score)	2.8 ± 3.5	4.0 ± 2.5	0.717
Depressive symptoms (Goldberg scale score)	1.8 ± 2.7	1.4 ± 2.5	0.718
Sleep quality (Athens insomnia scale score)	3.2 ± 3.4	7.3 ± 4.7	0.170
Self-care agency (Appraisal of Self-care Agency Scale)	58.8 ± 8.0	57.2 ± 8.6	0.302
Quality of life (Short Form-36 Health Survey)	102.8 ± 6.0	99.3 ± 8.0	0.046 *
Body-mass index (self-reported weight/height)	26.2 ± 4.7	27.5 ± 4.7	0.614
A healthy diet (Adherence to the Mediterranean diet scale score)	9.4 ± 2.0	9.6 ± 1.9	0.618
Physical activity (minutes/week based on the International Physical Activity Questionnaire)	620.0 ± 688.1	55.3 ± 90.8	0.008 *

Footnotes: MH: mental health disorder; AD: Alzheimer’s disease. * Statistically significant difference (*p* < 0.05).

## Data Availability

The data presented in this study are available on request for scientific purposes from the corresponding author.
